# Effect of virtual reality distraction on venipuncture pain in children in the emergency room

**DOI:** 10.15649/cuidarte.3385

**Published:** 2024-07-03

**Authors:** María Elizabeth Gómez-Neva, Karol Johanna Briñez-Ariza, Leidy Johana Ibañez-Rodriguez

**Affiliations:** 1 Pontificia Universidad Javeriana, Bogotá, Colombia. E-mail: m.gomezn@javeriana.edu.co Pontificia Universidad Javeriana Pontificia Universidad Javeriana Bogotá Colombia m.gomezn@javeriana.edu.co; 2 Universidad de Ciencias Aplicadas y Ambientales UDCA, Bogotá, Colombia. E-mail: kbrinez.a@udca.edu.co Universidad de Ciencias Aplicadas y Ambientales Universidad de Ciencias Aplicadas y Ambientales UDCA Bogotá Colombia kbrinez.a@udca.edu.co; 3 Hospital Universitario San Ignacio, Bogotá, Colombia. E-mail: leidy.ibanez@javeriana.edu.co Pontificia Universidad Javeriana Hospital Universitario San Ignacio Bogotá Colombia leidy.ibanez@javeriana.edu.co

**Keywords:** Virtual reality, Nursing, Childcare, Vascular Access Devices, Acute Pain, Realidad Virtual, Enfermería, Cuidado del Niño, Dispositivo de Acceso Vascular, Dolor Agudo, Realidade Virtual, Enfermagem, Cuidado da Crianga, Dispositivos de Acesso Vascular, Dor Aguda

## Abstract

**Introduction:**

Virtual reality (VR) as a distraction strategy has been used in healthcare centers; however, the evidence is inconsistent in demonstrating VR's effect on pain control during venipuncture.

**Objective:**

To describe the effect of VR on pain during the venipuncture process in children and adolescents in a private institution in Bogotá.

**Materials and methods:**

An unblinded, randomized, experimental study was conducted in the emergency room of a fourth-level care facility. The sample consisted of 46 children and adolescents between the ages of 7 and 14. Twenty- three were randomly assigned to the experimental group with a VR headset and 23 to the control group. The dependent variable ‘pain’ was measured before, during, and after venipuncture using the visual analog scale (VAS) of pain.

**Results:**

Perceived pain is different before and after the procedure; however, using the VR headset did not show any statistical or clinical differences during the venipuncture procedure.

**Discussion:**

Variables such as vital signs, venipuncture time, and follow-up were important in measuring symptoms such as pain at venipuncture.

**Conclusion:**

VR can be used by nurses; however, more research must be done to demonstrate its effect on pain control during venipuncture, considering a greater power of the study, type of pain, and variables such as family support and nurses’ time of experience.

## Resumen

**Introducción:**

La realidad virtual como estrategia de distracción es utilizada en los centros de atención en salud, sin embargo, la evidencia no es consistente en demostrar el efecto en el control del dolor durante el proceso de venopunción.

**Objetivo:**

Describir el efecto de la realidad virtual en el dolor durante el proceso de venopunción en niños y adolescentes, en una institución privada en la ciudad de Bogotá.

**Materiales y métodos:**

Estudio experimental aleatorizado sin cegamiento. Se realizó en el servicio de urgencias de una institución de IV nivel de atención. La muestra estuvo constituida por 46 niños y adolescente entre los 7 y 14 años. De forma aleatoria 23 estuvieron en el grupo experimental con visor de realidad virtual y 23 en el grupo control. La variable dependiente dolor fue medido antes, durante y después de la venopunción con la escala análoga para el dolor.

**Resultados:**

El dolor percibido es diferente antes o después del procedimiento, sin embargo, el uso de gafas de realidad virtual no mostro diferencias estadísticas ni clínicas durante el proceso de venopunción.

**Discusión:**

Variables como los signos vitales, tiempo de venopunción y acompañamiento mostraron ser importantes cuando se miden síntomas como el dolor en la venopunción.

**Conclusión:**

La realidad virtual puede ser usada por profesionales de enfermería, sin embargo, debe investigarse más para demostrar efecto en el control del dolor durante la venopunción, teniendo en cuenta un mayor poder del estudio, tipo de dolor y variables como acompañamiento de familiares, tiempo de experiencia del enfermero.

## Resumo

**Introdujo:**

A realidade virtual como estratégia de distragáo é utilizada em centros de saúde, porém as evidencias náo sáo consistentes em demonstrar o efeito no controle da dor durante o processo de pungáo venosa.

**Objetivo:**

Descrever o efeito da realidade virtual na dor durante o processo de pungáo venosa em crianzas e adolescentes, em uma instituido privada da cidade de Bogotá.

**Materiais e métodos:**

Estudo experimental randomizado sem cegamento. Foi realizado na sala de emergencia de uma instituido de atendimento de nivel IV. A amostra foi composta por 46 crianzas e adolescentes entre 7 e 14 anos. De forma aleatória 23 estavam no grupo experimental com visualizador de realidade virtual e 23 no grupo controle. A variável dependente dor foi mensurada antes, durante e após a pungáo venosa com escala analógica de dor.

**Resultados:**

A dor percebida é diferente antes ou após o procedimento, porém, o uso de óculos de realidade virtual náo apresentou diferengas estatísticas ou clínicas durante o processo de pungáo venosa.

**Discussao:**

Variáveis como sinais vitais, tempo de pungáo venosa e seguimento mostraram-se importantes na mensuragáo de sintomas como dor durante a pungáo venosa.

**Conclusao:**

A realidade virtual pode ser utilizada pelos profissionais de enfermagem, porém mais pesquisas devem ser feitas para demonstrar seu efeito no controle da dor durante a pungáo venosa, levando em consideragáo maior.

## Introduction

Venipuncture of a short peripheral venous line is a common procedure in nursing practice. It causes patients fear, anxiety, and pain and requires time and expertise for the practitioner to efficiently obtain venous access with a minimum of time and resources. However, healthcare professionals must be able to control pain before, during, and after the procedure to ensure an intervention with minimal risk and maximum safety for patients^1^.

There are factors that influence the perception of pain in children, and some articles based on aspects expressed by children and adolescents support this. The study by Pope et al. distinguishes the following factors: first, those inherent to a previous experience (vicarious experience), that is, what is expected to experience pain (expectations), and those inherent to a person’s social structure, which affect their attention, response, vulnerability, and opportunity to provide care. Second, pain can be classified according to its quality, anatomical location, cause, and meaning^2^. The present research did not approach pain from an experiential point of view; it approached pain only from a numerical perception, which limits the approach. Vejzovic et al.^3^ reported in their study that healthcare professionals ask children about the presence of pain; however, few of them use the Visual Analog Scale (VAS) on all occasions and allow children and adolescents to assess their pain based on their numerical perception.

Placing a short venous catheter is an art that requires knowledge and skill. Technique and assertiveness with children and their families are fundamental aspects of the professional experience, which in turn help to reduce the anxiety generated by the uncertainty of the procedure in terms of puncture success, pain, and functionality^4^.

The practice of care involves the integration of interventions, research, and humanization. In this sense, nursing care includes venipuncture as an intervention, research on the use of distraction, and humanization to reduce pain for those undergoing these painful interventions. For children, distraction means being entertained, thinking about something else, focusing on something else, and thinking that it will be interesting, thus shifting their thoughts away from what is happening^5^.

According to the literature, various distraction strategies have been used to distract infants and children. Swaddling and non-nutritive sucking are effective techniques for young infants. For preschoolers, coloring, drawing, or painting are common strategies. For schoolchildren and teenagers, distraction takes a more immersive approach through the use of audiovisual elements such as VR glasses and tablets. Other strategies include the use of pets, ice, and vibration^6^^, ^^7^.

Distraction allows attention to be diverted from pain, and VR has become a widely used tool in healthcare facilities. However, current evidence does not specifically demonstrate its effect on pain control during venipuncture. The purpose of this study is to provide scientific evidence on the effect of VR on pain control during venipuncture in children and adolescents.

## Materials and Methods

An unblinded, randomized clinical study was conducted between 2022 and 2023 in the pediatric emergency room of a private healthcare facility in Bogotá (Colombia). The population consisted of children and adolescents between the ages of 7 and 14 with a medical indication for venous access. Children and adolescents with an altered state of consciousness, impaired vision or use of glasses, history of failed venipuncture without the use of goggles for visual distraction during the current hospitalization, lack of consent of legal representatives or refusal to participate in the study, and clinically unstable were excluded from the study. The sample size was calculated with a type I error of 5% and 80% power, yielding a sample of 46 participants, 23 experimental and 23 controls. Assignment to the intervention or control group was randomized according to the last number of the patient's ID (if even, the patient was assigned to the experimental group; if odd, the patient was assigned to the control group).

### Intervention

The Samsung Gear VR^8^ headset was used, a visual distraction device that allows users to enjoy 2D, 3D, and 360-degree content by connecting it to a mobile device. It features a 101°/62mm (fixed) /10mm lens that provides a wide field of view, interpupillary distance, and eye relief to prevent eye strain during distraction. The device has gyroscopic and proximity sensors, allowing children and teenagers to visualize images in a near-real environment. It is compatible with mobile phones via USB or micro USB and includes a remote control for easy navigation through VR. This control can be operated with one hand, simplifying the intervention in this study. The controller also connects to a mobile device paired with the Gear VR via Bluetooth.

### Visual distraction game

The game used, Sky Night, is a free game for Android devices. To date, the effect of this game on pain control when played by children with VR headsets has not been studied or demonstrated. A pilot study found that this game does not require the use of the hands, as it can be controlled by the movement of the head alone, facilitating the venipuncture and the measurement of the variables in this study.

### Measurement instrument

Pain intensity was measured using a numeric visual analog scale (VAS), where 0 indicates no pain, and 10 indicates severe pain. In addition, a data collection form was designed that included variables characterizing the venipuncture process and variables related to vital signs. The authors of the scale mention that it has been widely used. It should be noted that the numerical VAS has no items; it only indicates pain in a scale from 0 to 10, so it is not common to perform validation based on internal consistency (Cronbach's alpha), which is the correlation between the items of an instrument^9^.

### Procedure

An attending pediatrician examined the child or adolescent and determined the relevance and indication for peripheral venous access as part of treatment. The legal representatives were informed of the purpose and objectives of the study to obtain their informed consent, as well as the participant's informed assent with the signature of two eyewitnesses. The participant was then randomized to the experimental or control group according to the participant’s identification number.

In the case of assignment to the experimental group, it was explained to the participants and parents or companions that they would wear the Samsung Gear VR headset and observe some images (stars) that they would have to join together to form another image. This process required movement of the participant's head and one hand to interact with theVR game. The visual distraction device was placed before venipuncture and removed after the procedure. The control group received venipuncture with the standard intervention. Nurses in both groups had one year or more experience with pediatric venipuncture. Data was collected over a 12-month period from 2022 to 2023.

Regardless of the group, vital signs and pain were measured before, during, and after the venipuncture procedure using a Mindray pediatric vital signs monitor. These data were recorded on paper forms and digitized weekly into an Excel database. Measurements and recordings occurred in the same pediatric emergency room, using the same monitors and forms for both groups.

### Dataset

The informed consent and data collection forms were organized in AZ binders and stored in the office of the principal investigator, MEGN. The information from these forms was entered into an Excel database on a weekly basis by two of the researchers, KJBA and LJI. All collected data, including data description, files, categories, and steps for reproducing the results, are available for free access and consultation in Mendeley Data^10^.

### Data analysis

The data were analyzed in the Python program. Descriptive statistics were used for categorical variables, and measures of central tendency were used for ratio variables. Hypothesis testing was performed using the mean difference test for continuous variables and the Chi-square (X2) test for categorical variables. A random forest with a 95% confidence level was used to obtain the model of variables that predict pain in pediatric or adolescent patients during the venipuncture process.

### Control of bias

Participants were randomized into experimental and control groups to minimize selection bias. Regarding confounding bias, this was addressed by including children and adolescents aged 7 to 14, as children younger than 7 and adolescents older than 14 have different responses to pain during medically invasive procedures.

### Ethical considerations

The project was submitted to and approved by the Institutional Research Ethics Committee.

## Results

**Figure 1:**
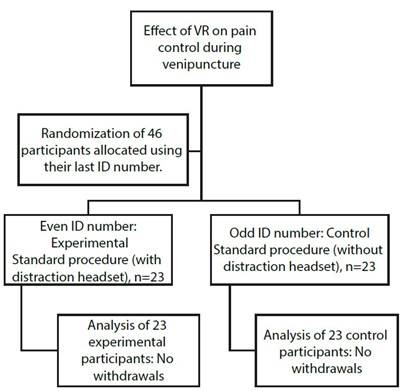
Participant flow diagram

The present experimental research study is reported according to the CONSORT statement (see Figure 1).

### Sample characteristics

The sample included children and adolescents with an average age of 11.2 years (± 3.5), comprising 60.14% females, with 76.40% accompanied by their mothers. Participants were most frequently cannulated on the dorsum of the hand (65.00%). The mean length of experience of the nurse performing the procedure was 7.58 ± 7.25 months. The average duration of the venipuncture procedure was 11.33 ± 13.04 minutes (Table 1).

**Table 1:** Study participant characteristics

Variable	Total (n=46)	Experimental (n=23)	Control (n=23)	p-value
Age, Mean ± SD	11.21 ± 3.50	10.82 ± 3.94	11.52 ± 3.04	0.50
Sex %(n)				0.05
Male	40.06(18)	50.01(11)	30.12(12)	
Femenino	60.14(28)	50.31(12)	70.11(16)	
Companion %(n)				0.98
Mother	76.40(35)	83.13(19)	70.15(16)	
Father	13.05(6)	9.42(2)	17.15(4)	
Grandparents	4.01(2)	4.01(1)	4.01(1)	
Aunts or uncles	4.00(2)	0.00(0)	9.02(2)	
Other	2.11(1)	4.21(1)	0.00(0)	
Venipuncture site %(n)				0.56
Back of the hand	65.00(31)	70.26(16)	60.01(15)	
Forearm	26.21(12)	26.21(6)	26.21(6)	
Dorsum of the foot	4.01(2)	4.01(1)	4.01(1)	
Thumb	5.41(1)	0.00(0)	10.01(1)	
Nurse experience in years, mean ± SD	7.58±7.25	10.78±6.76	11.78±7.52	0.14
Venipuncture time in minutes, mean ± SD	11.33 ±13.04	10.90 ±11.21	11.81 ±15.21	0.79
Time to successful venipuncture in minutes, mean ± SD	261.10±299	207.13±211.77	312.57±261.36	0.24
SBP, mean ± SD	106.41±11.91	107.04±10.55	105.82±13.29	0.74
DBP, mean ± SD	70.6±11.06	72.13±9.94	68.86±12.18	0.63
MAP, mean ± SD	81.82±10.67	82.39±9.91	81.86±11.57	0.96
HR, mean ± SD	96.62±20.55	98.50±18.44	96.04±22.80	0.85
Temperature, mean ± SD	36.90±0.71	36.58±0.64	36.57±0.78	0.85
Oxygen saturation, mean ± SD	93.97±3.75	94.08±3.24	94.01±4.26	0.96

*SBP: Systolic blood pressure, DBP: Diastolic blood pressure, MAP: mean arterial pressure, HR: Heart rate, p-value: The mean difference test and the Chi-square (X2)*

### Effect of VR on vital signs during the venipuncture process.

No statistically significant differences were found between the intervention and control groups in terms of vital signs (heart rate, blood pressure, respiratory rate, and oxygen saturation) before and after the intervention (Table 2).

**Table 2:** Effect of VR on vital signs before and after venipuncture

	Experimental group			Control group		
Before	After	p-value	Before	After	p-value
SBP	107.04 ±10.31	109.86±9.72	0.62	105.82±13.29	108.69±11.53	0.43
DBP	72.13±10.24	70.56±12.05	0.42	69.86±12.18	69.13±9.54	0.81
MAP	82.39±10.19	83.48±9.29	0.65	81.86±11.57	83.04±9.49	0.71
HR	96.50±18.37	100.6±19.43	0.62	96.04±22.80	100.39±23.60	0.52
Temperature	36.58±0.64	36.70±0.59	0.93	36.57±0.68	36.63±0.74	0.80
Oxygen saturation	94.08±3.23	94.86±2.95	0.30	94.00±4.26	94.95±3.22	0.39
Pain	2.40±3.28	4.69±4.19	0.005	4.13±3.84	6.39±3.75	0.049

*SBP: Systolic blood pressure, DBP: Diastolic blood pressure, MAP: mean arterial pressure, HR: Heart rate, p-value: The dependent mean difference test*

### Effect of virtual reality on pain control during venipuncture

Pain was measured before, during, and after the procedure. Differences were observed between the intervention and control groups before the procedure (P=0.039), but no differences were observed after the venipuncture.

### Pain related variables

**Figure 2:**
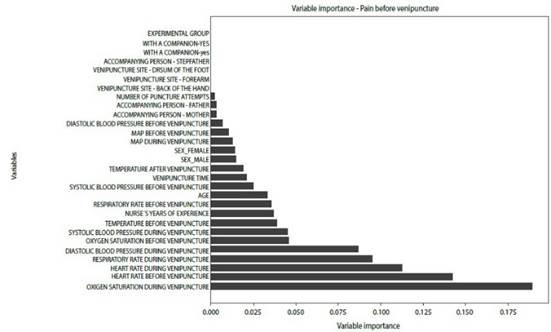
Overall importance of pain-related variables prior to venipuncture in the experimental group

**Figure 3:**
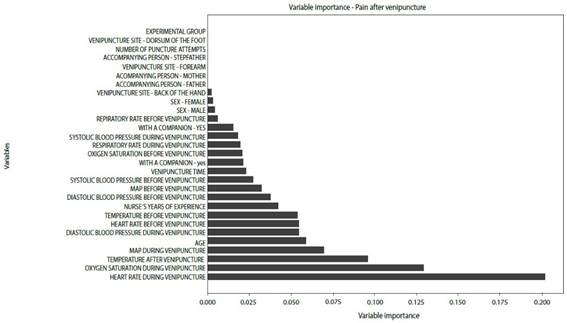
Overall importance of pain-related variables after venipuncture in the experimental group

A model was created to identify the most important variables influencing pain before and after venipuncture in the experimental group. Variables such as heart rate, respiratory rate, oxygen saturation, and blood pressure before and during the procedure were important markers of pain during venipuncture in children and adolescents. Variables such as the nurse's years of experience, the participant's age, and the father's presence were important before the procedure. Other variables, such as the duration of the venipuncture and the mother's presence, were important after the procedure (Figures 2 and 3).

*MAP: meanarterial pressure*

*MAP: meanarterial pressure*

## Discussion

An unblinded experimental study was conducted on children and adolescents to evaluate the effect of virtual reality on the venipuncture process. Venipuncture has become a common and painful procedure used to diagnose and treat medical conditions. Venipuncture causes objective changes in vital signs, affecting both the physical and emotional aspects of the body's sympathetic or parasympathetic physiological response. As a result, the venipuncture process is impeded by vasoconstriction or vasodilation, as well as skin sweating, which complicates the fixation of peripheral venous access^11^. The results of this study showed that variables such as heart rate, blood pressure, and oxygen saturation before and during the venipuncture procedure are important when it comes to symptoms caused by the procedure, such as pain. However, they did not show any differences between the VR intervention group and the control group. Other variables, such as the mother's or father's presence, are variables that showed different importance before and after the procedure, which may influence anxiety and pain during venipuncture.

The pain assessment should be appropriate to the participant's age group, and the intervention should meet the specific needs of that age group^12^^, ^^13^. While assessing pain with the numerical pain scale, the study could have used the VAS with pictures to facilitate response and used a game that was not previously reported to be effective on pain and did not respond to participants’ preferences.

In the present study, no difference in the perception of pain was found between the use or non-use of VR during and after the venipuncture procedure. However, several authors^12^^, ^^14^^, ^^16^ have evaluated fully immersive virtual reality in visual and auditory aspects and identified it as a protective factor in reducing pain, distress, and anxiety during venipuncture. Moadad et al.^17^ demonstrated that using the Buzzy device, which combines vibration and cold, was an effective intervention for reducing pain ratings. It is important to note that the Wong-Baker Faces Pain Rating Scale was used in the study mentioned above, which may have allowed children to more easily identify with the pain they experience when viewing facial expressions, thus facilitating assessment during venous catheter insertion. The evidence is consistent that studies are still needed to determine the effect of this intervention on pain control for various painful procedures^18^, which is consistent with the results of our study.

In the present study, pain was rated by the children or adolescents themselves using a numerical scale before, during, and after venipuncture. However, pain before venipuncture may have had a different etiology, which is why the emergency room visit occurred. The difference in pain measurements between the intervention and control groups before venipuncture (P=0.039) may be due to this confounding factor, as there were no significant differences in pain measurements during or after venipuncture (P=0.174). It is acknowledged that VR is unlikely to be the primary treatment for managing pain that prompts emergency room visits. This aligns with a study that found low certainty in the effectiveness of VR distraction for children with acute pain. However, there is a case report that suggests that VR, unlike many other treatments, supports the reduction of severe refractory pain syndrome in adolescents^14^.

This study used VR as a distraction tool before, during, and after the procedure. During this time, participants had the opportunity to interact with a game that involved movements of the head and one hand to respond to the game's challenges. The game was selected in advance by the researchers; however, evidence indicates that the distraction should align with participants' preferences, including colors, images, music, and other factors that may influence their response to the virtual reality intervention^13^. The use of movement, sensation, and thought directs the individual's attention to the game, image, music, or video, possibly reducing the perception of painful stimuli that are not visually perceived. These aspects should be considered in future studies that attempt to distract not only one but several senses (hearing, smell, touch) simultaneously to elicit a motor or cognitive response that alleviates an unpleasant sensation.

In addition, the present study was conducted in a pediatric emergency setting, where many variables are difficult to control. The VR headset used was not fully immersive, providing only visual distraction and no sound to facilitate auditory immersion. This contrasts with other studies conducted in similar settings and with children of similar ages, which have shown more favorable results^12^.

For children, distraction means being entertained, thinking about something else, concentrating on something else, thinking it will be interesting, and shifting their thoughts away from what is currently happening^5^. Studies such as that of Sánchez et al.^19^ show positive results when distraction is incorporated into the care of the pediatric population, benefiting dimensions such as physical, psychological, social, and immune health. VR is one distraction strategy that may or may not be immersive. Our study only evaluated the effect of VR with a video game using a VR headset that is considered non-immersive^20^, and no immersive methods were used. There is evidence that immersive methods have an effect on pain in pediatric patients, as reported by Gupta et al.^21^ They believe that devices such as VR helmets alter the ability to perceive pain and respond to noxious external stimuli.

According to Sánchez et al.^19^, children and adolescents may present with psychosomatic symptoms, such as abdominal, joint, and muscle pain, among others, due to familial, genetic, relational, or dysfunctional situations, such as educational errors, parental overprotection, family pressure, excessive demands on the child, abuse, and insecure attachment^21^^, ^^22^. This study did not consider variables such as the companion's influence on the participant's care, nor did it assess whether the relationship between them was conflicted. This could have influenced the participant's assessment of pain, especially in cases of overprotection or detachment. In comparison to the present study, the research by Ferraz et al.^12^ used the Perioperative Adult and Child Behavioral Interaction Scale (PACBIS), which identifies behaviors in children and their parents and defines parental behavior as reactive or blocking, considered negative, or proactive, considered positive.

Nurses are constantly striving to perform safe and effective procedures with minimal pain. Evidence suggests that nonpharmacologic interventions are effective in controlling pain during venipuncture^23^, and the use of distraction for pain control has been studied extensively^23^^, ^^24^. However, it has been observed that painful procedures are still performed without alleviating the symptom of "procedural pain," which is contrary to nursing care duties. This is due to methodological limitations, inconsistencies, and inaccuracies in the studies that affect the quality of the evidence. The results of our research highlight the need for studies on the effect of immersive VR on painful procedures in children and adolescents. Thus, evidence-based protocols using distraction therapies, such as VR, could be developed to reduce the pain and anxiety associated with these procedures in children, promoting safer and more efficient care practices^25^.

The present study had limitations related to the control of variables such as pain and anxiety associated with the admission diagnosis and venipuncture time, which were critical in comparing the experimental and control groups before and after venipuncture. Another limitation was the low number of venipunctures per patient, as the short peripheral venous access should only be changed if there is a sign of complication^11^. Despite the limitations described above, the study reached the estimated sample size for the proposed objective.

## Conclusion

Non-immersive virtual reality showed no effect on pain control during venipuncture in children and adolescents. The results show that further research is needed to demonstrate the effect and thereby maximize the safety and quality of our care processes. Variables such as preprocedural anxiety, the presence of family members, and game preferences are identified as confounding variables in estimating the effect. Sensory distraction to control symptoms such as pain should be evaluated using different senses, such as sight, hearing, and even smell.

## References

[1] Gómez-Neva E, Bayona JG, Rosselli D (2015). Flebitis asociada con accesos venosos periféricos en niños: revisión sistemática de la literatura. Infectio.

[2] Pope N, Tallón McConigley R, Leslie G Wilson S (2017). Experiences of acute pain in children who present to a healthcare facility for treatment: a systematic review of qualitative evidence. JBI Evidence Synthesis.

[3] Vejzovic V, Bozic J, Panova G, Babajic M, Bramhagen A (2020). Children still experience pain during hospital stay: a cross-sectional study from four countries in Europe. BMC Pediatrics.

[4] Gerçeker GÖ, Bektaş M, Aydinok Y, Ören H, Ellidokuz H, Olgun N (2021). The effect of virtual reality on pain, fear, and anxiety during access of a port with huber needle in pediatric hematology oncology patients: Randomized controlled trial. Eur J Oncol Nurs.

[5] Briñez Ariza KJ, Gomez-Neva ME (2020). Distraction preferences of children with cancer. Qualitative Research in Health: advances and challenges.

[6] Sampson J, Allbright R (2019). Distraer a los pacientes pediátricos durante los procedimientos dolorosos. Nursing.

[7] Gad R, Wilson M (2023). Can we safely manage pain using virtual reality (VR). Pain Manag Nurs.

[8] Samsung (2023). Manual de Usuario SM-R322.

[9] Tavakol M, Dennick R (2011). Making sense of Cronbach's alpha. Int J Med Educ.

[10] Gómez ME, Briñez KJ Ibañez L (2023). Efecto de la distracción con realidad virtual en el dolor por venopunción en niños de urgencias. Mendeley Data.

[11] Gómez-Neva E, Rondon Sepulveda M, Buitrago-Lopez A (2022). Lifespan of peripheral intravenous short catheters in hospitalized children: A prospective study. Journal of Vascular Access.

[12] Ferraz-Torres M, Soto-Ruiz N, Escalada-Hernández P, García-Vivar C, San Martín-Rodríguez L (2023). Can virtual reality reduce pain and anxiety in pediatric emergency care and promote positive response of parents of children? A quasi-experimental study. IntEmergNurs.

[13] Plaza Sánchez L, Gómez Galán R (2015). Efectividad en la aplicación de un método de distracción audiovisual en niños durante la vacunación. Rev cubana Enfermería.

[14] Sørensen JCH, Vlachou M, Milidou I, Knudsen AL, Meier K (2023). Virtual Reality Treatment of Severe Neuropathic Pain in an Adolescent Child: A Case Report. A Pract.

[15] Sandoval-González A, Guzmán-Saldaña RME, Lerma-Talamantes A, Arrieta-Villareal JL (2023). Realidad Virtual y dolor en niños y adolescentes con cáncer: una revisión sistemática. Educación Y Salud Boletín Científico Instituto De Ciencias De La Salud Universidad Autónoma Del Estado De Hidalgo.

[16] Lambert V, Boylan P, Boran L, Hicks P, Kirubakaran R, Devane D (2020). Virtual reality distraction for acute pain in children. Cochrane Database Syst Rev.

[17] Moadad N, Kozman K, Shahine R, Ohanian S, Badr LK (2016). Distraction Using the BUZZY for Children During an iV Insertion. J PediatrNurs.

[18] Herrero Arnedo N, Almudéver Campo L (2020). Revisión sistemática en la literatura científica del uso de la realidad virtual como tratamiento de los trastornos psicosomáticos. Tesis Psicológica.

[19] Sánchez MA, Córdova GK, Vásquez MP, Briñez KJ (2022). Resultados de distracción para el cuidado en oncología pediátrica desde la evidencia de enfermería: revisión integrativa. Enferm. glob.

[20] Gad R, Wilson M (2023). Can we safely manage pain using virtual reality (VR). Pain Management. Nursing.

[21] Gupta A., Scott K, Dukewich M (2018). Innovative technology using virtual reality in the treatment of pain: Does it reduce pain via distraction, or is there more to it?. Pain Medicine.

[22] Sánchez Boris IM (2020). Los trastornos psicosomáticos en el niño y el adolescente. MEDISAN.

[23] Bergomi P, Scudeller L, Pintaldi S, Dal Molin A (2018). Efficacy of Non-pharmacological Methods of Pain Management in Children Undergoing Venipuncture in a Pediatric Outpatient Clinic: A Randomized Controlled Trial of Audiovisual Distraction and External Cold and Vibration. J Pediatr Nurs.

[24] Birnie KA, Noel M, Chambers CT, Uman LS, Parker JA (2018). Psychological interventions for needle- related procedural pain and distress in children and adolescents. Cochrane Database of Systematic Reviews.

[25] Ozalp Gerçeker G, Ayar D, Ozdemir EZ, Bektaş M (2020). Effects of virtual reality on pain, fear, and anxiety during blood draw in children aged 5-12 years old: A randomised controlled study. J Clin Nurs.

